# Prevalence and comorbidity burden of clinical obesity in US adults

**DOI:** 10.1186/s12889-026-27457-3

**Published:** 2026-04-29

**Authors:** Tuo Xiao, Le Yang, Congying Fan, Yanna Dou, Jin Shang

**Affiliations:** 1 Department of Nephrology, The First Affiliated Hospital of Zhengzhou University, Zhengzhou, Henan 450052 China; 2 The First Clinical School of Zhengzhou University, Zhengzhou University College of Medicine, Zhengzhou, Henan 450052 China

**Keywords:** Clinical obesity, National Health and Nutrition Examination Survey, Comorbidity

## Abstract

**Background:**

This study aimed to estimate the prevalence and comorbidity burden of clinical obesity among US adults.

**Methods:**

Analyzing data from 53,333 US adults (NHANES, 1999–2018), we stratified participants into no obesity, preclinical obesity, and clinical obesity based on BMI, waist circumference, and presence of organ dysfunction or functional limitations. Mortality risks were assessed via survey-weighted Cox proportional hazards regression, Fine-Gray subdistribution hazard model, and Kaplan–Meier curves. Latent class analysis (LCA) identified distinct comorbidity clusters.

**Findings:**

Overall obesity prevalence rose from 46.7% to 61.7%, with clinical obesity (from 34.6% to 46.6%) constituting the dominant type. Clinical obesity was associated with significantly higher risks of all-cause (HR 1.46, 95% CI 1.29–1.66) and cardiovascular (HR 1.65, 95% CI 1.29–2.10) mortality versus preclinical obesity. Within clinical obesity, individuals with ≥ 5 comorbidities had an increased all-cause mortality 3.0-fold (25.4 vs 7.8 per 1000 person-years) and cardiovascular mortality 3.4-fold (5.1 vs 1.6 per 1000 person-years) compared to a single comorbidity. LCA revealed three distinct comorbidity clusters with prognostic heterogeneity; the renal dysfunction-dominant cluster exhibited the poorest survival.

**Conclusion:**

The expanding US obesity epidemic is predominantly characterized by a growing burden of high-risk clinical obesity, signifying a shift towards more severe disease phenotypes. Accurate comorbidity quantification in clinical obesity is crucial for refining prognostic assessment and optimizing treatment strategies.

**Supplementary Information:**

The online version contains supplementary material available at 10.1186/s12889-026-27457-3.

## Introduction

Over the past three decades, the prevalence of obesity has generally increased worldwide, and no country has successfully curbed this upward trend, resulting in a substantial increase in its associated global burden [1]. Moreover, obesity serves as a significant risk factor for a broad spectrum of chronic conditions, including cardiovascular and respiratory diseases, musculoskeletal injuries, and even malignant tumors. It is increasingly recognized as a primary factor contributing to premature death [2]. Consequently, its prevention and management have become a critical priority across public health, clinical medicine, and economic sectors [3]. Given its status as one of the most modifiable determinants of future health, there is an urgent need to elevate our conceptual understanding of obesity. This mandates the implementation of more vigorous and precise mitigation strategies to counteract its unparalleled threat to morbidity and mortality at local, national, and global scales [1].

Despite this urgency, current assessment strategies relying solely on body mass index (BMI) frequently yield insufficient individual health profiling, thereby compromising the efficacy of both clinical care and public health policy [4]. To rectify this limitation, the Lancet Diabetes & Endocrinology Commission established a refined diagnostic paradigm aimed at enhancing the clinical assessment of obesity status. This framework categorizes obesity into clinical obesity and preclinical obesity based on clinical treatment needs, aiming to underscore the urgency of addressing its complications. Particularly, it emphasizes that clinical obesity should be recognized as a disease on par with other major chronic diseases [5]. Recent epidemiological evidence reinforces this conceptual shift, demonstrating significant prognostic divergence between these two phenotypes, thereby validating the need for a more granular diagnostic approach [6].

Nevertheless, a comprehensive understanding of the clinical course of clinical obesity is essential for informing precision public health policies, yet research specifically characterizing this high-risk phenotype remains scarce. To address this gap, we analyzed two decades of nationally representative survey data from the National Health and Nutrition Examination Survey (NHANES). This study aimed not only to delineate the prevalence and comorbidity burden of clinical obesity using the newly proposed diagnostic framework but also to systematically evaluate its prognostic implications. Crucially, we sought to refine risk stratification within this heterogeneous population by determining how both the cumulative burden and the specific nature of comorbidities differentially influence mortality outcomes.

## Methods

### Study population

Data were derived from the NHANES (1999–2018), a continuous program utilizing a sophisticated, multi-stage probability sampling approach designed to yield nationally representative estimates for the US civilian population [7]. Of the 101,316 initial candidates, we sequentially excluded individuals aged < 19 years (*N =* 44,207), those with missing anthropometric measurements, BMI and waist circumference (*N =* 3670), and those without mortality records (*N =* 106). The final analytic cohort consisted of 53,333 adults (Supplemental Fig. 1).

### Study variables

Sociodemographic characteristics were analyzed, including age, sex, race/ethnicity, education level, and the income-to-poverty ratio (PIR). Anthropometric assessments comprised height, waist circumference, and BMI. Lifestyle factors included smoking status, defined as a lifetime history of smoking ≥ 100 cigarettes, and alcohol consumption, categorized as never (< 12 drinks annually), former (a history of ≥ 12 drinks in a year but no consumption in the past 12 months), and current (any drinking within the past 12 months). Diet quality was assessed using the Healthy Eating Index-2015 (HEI-2015), a validated measure of overall diet quality and adherence to the 2015–2020 Dietary Guidelines for Americans [8]. Dietary intake data were collected from participants through two 24-h dietary recall interviews conducted in NHANES.

Laboratory evaluations included serum or plasma measurements of platelets, glycated hemoglobin, fasting plasma glucose, triglycerides, high‐density lipoprotein cholesterol, and liver enzymes (alanine aminotransferase, aspartate aminotransferase, and gamma-glutamyltransferase). The estimated glomerular filtration rate (eGFR) was calculated based on serum creatinine levels using the 2021 CKD-EPI equation. Urine albumin-to-creatinine ratio (ACR) was determined by dividing albumin concentration by creatinine concentration in urine. Clinical history of obesity-related comorbidities and functional impairments was ascertained, encompassing medication use, chronic obstructive pulmonary disease (including emphysema), heart failure, urinary incontinence, and limitations in physical functioning.

### Definitions of obesity status

Following the diagnostic framework proposed by the Lancet Diabetes & Endocrinology Commission [5], obesity was defined if participants met at least one of the following three criteria: (1) BMI ≥ 40 kg/m^2^; (2) elevated BMI combined with either central adiposity or waist-to-height ratio > 0.5; (3) central adiposity combined with waist-to-height ratio > 0.5. Elevated BMI was defined as ≥ 27.5 kg/m^2^ for Asian populations and ≥ 30 kg/m^2^ for non-Asian populations. Central adiposity was defined using race-specific waist circumference thresholds: > 90 cm in men and > 80 cm in women for Asian participants, and > 102 cm in men and > 88 cm in women for non-Asian participants. Participants meeting obesity definitions were subsequently stratified by health status: those exhibiting functional limitations or signs of organ dysfunction were classified as clinical obesity, whereas the remainder were designated as preclinical obesity (Supplemental Table 1).

### Statistical analysis

All analyses incorporated NHANES' sample weights, strata, and primary sampling units to account for its complex multistage probability sampling design. Baseline characteristics were stratified by obesity phenotypes (No obesity, Preclinical obesity, and Clinical obesity). Descriptive statistics included means ± standard deviation (SD) for continuous variables and numbers (percentages) for categorical variables. Baseline characteristics across the three groups were compared using one-way analysis of variance (ANOVA) for continuous variables and the chi-square test for categorical variables. To reduce selection bias and maintain statistical power, missing values for essential covariates—including education, PIR, smoking status, alcohol intake, cancer history, and HEI-2015—were imputed using Multiple Imputation by Chained Equations (MICE).

Survey-weighted prevalence was calculated for each survey cycle, accounting for the complex sampling design and applying NHANES examination weights. Weighted Cox regression models were used to assess the association between the obesity phenotype and both all-cause and cardiovascular mortality. Three models were constructed to control for potential confounders: Model 1, unadjusted model; Model 2, adjusted for age, sex, and race/ethnicity; Model 3, adjusted for age, sex, race/ethnicity, PIR, education level, smoking, alcohol consumption, cancer history, and HEI-2015. Multiplicative interaction terms between obesity phenotypes and continuous age were incorporated into the fully adjusted models, allowing for the estimation of age-specific hazard ratios (clinical obesity vs. no obesity) at ages 30, 45, 60, and 75 years via linear combinations of coefficients.

Sensitivity analyses were conducted using multivariable-adjusted Cox proportional hazards models to assess the robustness of mortality risk. To address the competing risk of cardiovascular mortality, the Fine-Gray subdistribution hazard model was employed. Cumulative incidence functions (CIFs) were estimated and compared across groups using Fine-Gray test.

To explore heterogeneity within clinical obesity, latent class analysis (LCA) was performed on participants with complete comorbidity data. Model selection was guided by multiple criteria, including the minimum bayesian information criterion (BIC), akaike information criterion (AIC), entropy, average posterior probability, clinical interpretability, and sample size (with each group accounting for > 5%). Participants were classified into corresponding clusters based on the highest posterior probability (Supplementary Table 3). Kaplan–Meier survival curves were generated to visually compare survival differences among the identified clusters. All analyses were completed using R software (version 4.5.1). A two-sided *P* value < 0.05 was considered statistically significant.

## Results

### Participants characteristics

This study included 53,333 participants, and baseline characteristics are presented in Table 1, stratified by obesity phenotype. Compared with the no-obesity and preclinical obesity groups, participants with clinical obesity were significantly older (mean 53.6 years) and exhibited the lowest socioeconomic status, characterized by lower income and education levels. Demographically, Mexican Americans were disproportionately represented in this group. Regarding lifestyle behaviors, although this group reported the lowest current alcohol consumption (47.7%), the prevalence of lifetime smoking remained substantial (46.3%). Most critically, the clinical obesity cohort faced the poorest prognosis: all-cause (17.8%) and cardiovascular mortality (4.8%) rates were more than twofold higher than those in the preclinical obesity group, highlighting the severe compounding impact of the comorbidities on survival (all *P <* 0.001).

**Table 1:** Baseline characteristics of participants

Characteristics	Total (*N =* 53,333)	No obesity (*N =* 23,520)	Preclinical obesity (*N =* 7104)	Clinical obesity (*N =* 22,709)	*P* value
Age (years)	48.4 (18.7)	45.1 (19.4)	43.2 (16.5)	53.6 (17.4)	< 0.001
Sex					< 0.001
Male	25,739 (48.3%)	14,176 (60.3%)	2448 (34.5%)	9115 (40.1%)	
Female	27,594 (51.7%)	9344 (39.7%)	4656 (65.5%)	13,594 (59.9%)	
Race/ethnicity					< 0.001
Mexican American	9564 (17.9%)	4052 (17.2%)	1249 (17.6%)	4263 (18.8%)	
Other Hispanic	4373 (8.2%)	1967 (8.4%)	516 (7.3%)	1890 (8.3%)	
Non-Hispanic White	23,097 (43.3%)	10,253 (43.6%)	2933 (41.3%)	9911 (43.6%)	
Non-Hispanic Black	11,378 (21.3%)	4878 (20.7%)	1717 (24.2%)	4783 (21.1%)	
Other races	4921 (9.2%)	2370 (10.1%)	689 (9.7%)	1862 (8.2%)	
Education Level					< 0.001
Less than high School	14,495 (27.2%)	6301 (26.8%)	1479 (20.8%)	6715 (29.6%)	
High School	12,411 (23.3%)	5286 (22.5%)	1571 (22.1%)	5554 (24.5%)	
More than high School	26,427 (49.6%)	11,933 (50.7%)	4054 (57.1%)	10,440 (46.0%)	
PIR	2.49 (1.62)	2.51 (1.64)	2.68 (1.66)	2.40 (1.58)	< 0.001
BMI (kg/m^2^)	28.8 (6.82)	24.0 (3.44)	31.6 (5.85)	32.9 (6.58)	
Waist circumference (cm)	98.3 (16.2)	85.3 (9.15)	104 (12.5)	109 (13.5)	
Waist-to-height ratio	0.59 (0.10)	0.51 (0.05)	0.63 (0.07)	0.67 (0.08)	
HEI-2015	52.7 (13.3)	52.7 (13.5)	52.8 (13.1)	52.7 (13.2)	0.918
Alcohol consumption					< 0.001
Never	15,102 (28.3%)	5687 (24.2%)	2073 (29.2%)	7342 (32.3%)	
Former	9383 (17.6%)	3885 (16.5%)	970 (13.7%)	4528 (19.9%)	
Current	28,848 (54.1%)	13,948 (59.3%)	4061 (57.2%)	10,839 (47.7%)	
Smoking					< 0.001
Yes	24,037 (45.1%)	10,851 (46.1%)	2673 (37.6%)	10,513 (46.3%)	
No	29,296 (54.9%)	12,669 (53.9%)	4431 (62.4%)	12,196 (53.7%)	
Hypertension	10,308 (19.3%)	3610 (15.3%)	0 (0%)	6698 (29.5%)	< 0.001
Hyperglycemia	14,979 (28.1%)	5364 (22.8%)	0 (0%)	9615 (42.3%)	< 0.001
Dyslipidemia	18,958 (35.5%)	5579 (23.7%)	0 (0%)	13,379 (58.9%)	< 0.001
Heart failure	1663 (3.1%)	535 (2.3%)	0 (0%)	1128 (5.0%)	< 0.001
Respiratory diseases	1453 (2.7%)	566 (2.4%)	0 (0%)	887 (3.9%)	< 0.001
Liver dysfunction	1946 (3.6%)	220 (0.9%)	0 (0%)	1726 (7.6%)	< 0.001
Renal dysfunction	3271 (6.6%)	1142 (5.3%)	0 (0%)	2129 (9.8%)	< 0.001
Urinary incontinence	3979 (7.5%)	1186 (5.0%)	0 (0%)	2793 (12.3%)	< 0.001
Musculoskeletal diseases	1837 (3.4%)	540 (2.3%)	0 (0%)	1297 (5.7%)	< 0.001
Limitations of daily activities	3768 (7.1%)	1292 (5.5%)	0 (0%)	2476 (10.9%)	< 0.001
Cancer history	4768 (8.9%)	1814 (7.7%)	455 (6.4%)	2499 (11.0%)	< 0.001
All-cause mortality	7967 (14.9%)	3454 (14.7%)	461 (6.5%)	4052 (17.8%)	< 0.001
Cardiovascular mortality	2032 (3.8%)	830 (3.5%)	103 (1.4%)	1099 (4.8%)	< 0.001
Follow-up time (months)	117 (66.6)	122 (67.5)	123 (67.2)	110 (64.9)	< 0.001

Data are presented as mean (SD) for continuous variables and n (%) for categorical variables. Group differences were assessed using one-way ANOVA and chi-square tests, respectively.

*Abbreviations*: *PIR* Poverty-income ratio, *BMI* Body mass index, *HEI-2015* Healthy Eating Index-2015.

### Prevalence of obesity phenotypes across NHANES survey cycles

**Fig. 1:**
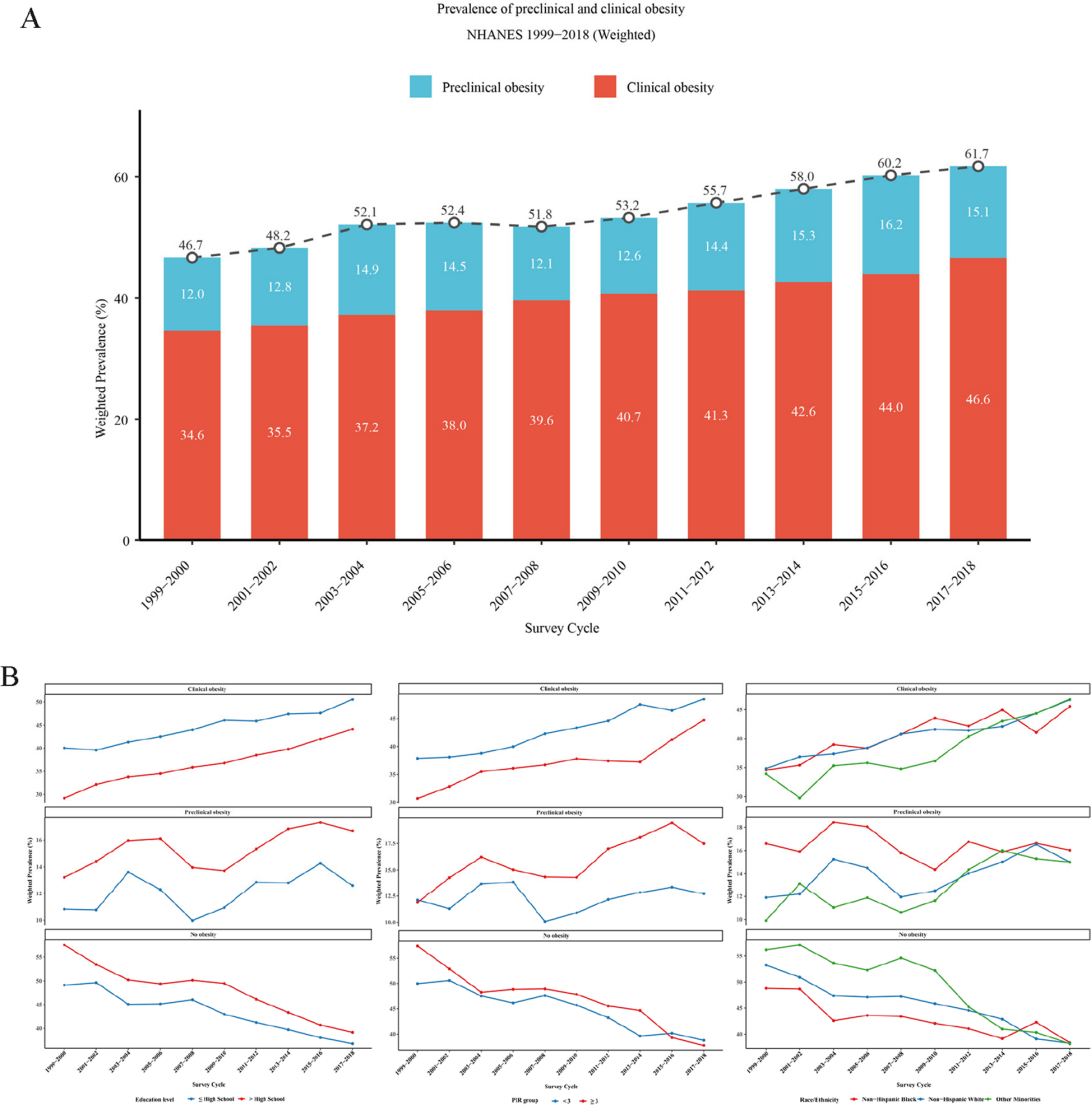
Prevalence of obesity phenotypes across NHANES survey cycles. **A** Weighted prevalence of preclinical and clinical obesity across NHANES Survey Cycles; **B** Weighted prevalence of obesity phenotypes stratified by education level, income, and race/ethnicity across NHANES survey cycles. Education level: ≤ high school and > high school; PIR group: < 3 and ≥ 3; Race/ethnicity: Non-Hispanic Black, Non-Hispanic White and Other Minorities (including Mexican American, Other Hispanic, and Other races)

Based on weighted data from nationally representative surveys conducted from 1999 to 2018 (Fig. 1A), the overall prevalence of obesity rose from 46.7% to 61.7%, with clinical obesity consistently constituting the dominant type. This upward trajectory was largely attributable to clinical obesity: its prevalence increased significantly from 34.6% to 46.6%, while the prevalence of preclinical obesity rose modestly (from 12.0% to 15.1%). These findings indicate a growing burden of obesity and highlight a shift towards more severe, comorbidity-associated obesity.

To further examine health disparities, we stratified patterns in obesity phenotypes by education, income, and race/ethnicity (Fig. 1B). Individuals with lower education (≤ high school) and lower income (PIR < 3) consistently exhibited a higher prevalence of clinical obesity, indicating a disproportionate burden in these disadvantaged groups. In contrast, those with higher education (> high school) and higher income (PIR ≥ 3) showed a higher prevalence of preclinical obesity, while the overall prevalence of obesity remained largely comparable. Obesity phenotypes across racial/ethnic groups did not show substantial differences.

### Association between clinical obesity and mortality risk

**Fig. 2:**
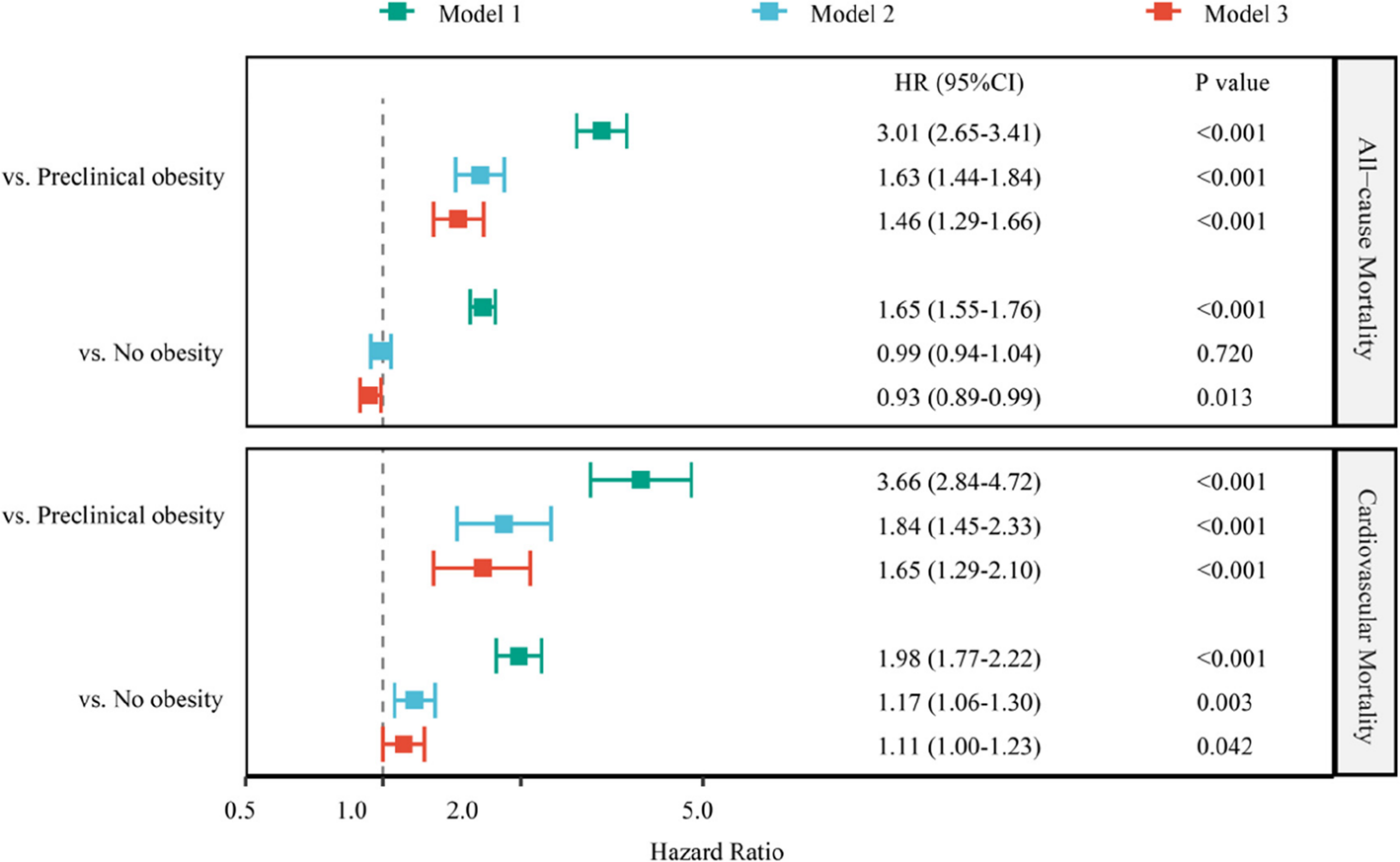
All-cause and cardiovascular mortality of clinical obesity compared with no obesity and preclinical obesity from Cox proportional hazards models. Model 1, unadjusted model; Model 2, adjusted for age, sex, and race/ethnicity; Model 3, adjusted for age, sex, race/ethnicity, PIR, education, smoking, alcohol consumption, cancer history, and HEI-2015. Median follow-up time: 112 months

Compared with the preclinical obesity group, the mortality risk of clinical obesity participants increased significantly in all models (Fig. 2). In the fully adjusted model (Model 3), clinical obesity increased the all-cause mortality risk by 46% (HR = 1.46, 95% CI: 1.29–1.66; *P <* 0.001), and the cardiovascular mortality risk by 65% (HR = 1.65, 95% CI: 1.29–2.10; *P <* 0.001), indicating that obesity with comorbidities confers a significantly elevated mortality risk.

Compared with the no obesity group, clinical obesity was significantly associated with a higher risk of all-cause mortality (HR = 1.65, 95% CI: 1.55–1.76; *P <* 0.001) and cardiovascular mortality (HR = 1.98, 95% CI: 1.77–2.22; *P <* 0.001) in the unadjusted model (Model 1). However, after fully adjusting for confounding factors (Model 3), while cardiovascular mortality risk remained elevated (HR = 1.11, 95% CI: 1.00–1.23; *P =* 0.042), the overall risk of all-cause mortality associated with clinical obesity shifted to a marginally significant inverse relationship (HR = 0.93, 95% CI: 0.89–0.99; *P =* 0.013) compared to the no obesity group. Further investigation, presented in Supplementary Table 2, revealed a significant interaction between obesity and age for both all-cause (P for interaction < 0.001) and cardiovascular mortality (*P* for interaction* =* 0.003). Using the linear combination of coefficients to estimate age-specific hazard ratios (clinical obesity vs. no obesity), we observed a consistent decline in the relative risk of clinical obesity with increasing age for both outcomes. Specifically, for all-cause mortality, the HR was 1.37 at age 30 and 1.17 at age 45, decreasing to 1.00 at age 60, and further to 0.85 at age 75, suggesting a potential protective association in older adults. A similar pattern was observed for cardiovascular mortality, with HRs of 2.21 at age 30 and 1.70 at age 45, attenuating to 1.30 at age 60, and becoming non-significant (HR = 1.00) by age 75. These interaction analyses indicate that the adverse impact of clinical obesity on mortality is predominantly concentrated in younger and middle-aged adults, with the relative risk diminishing in later life stages.

### Sensitivity and competing risk analyses

**Fig. 3:**
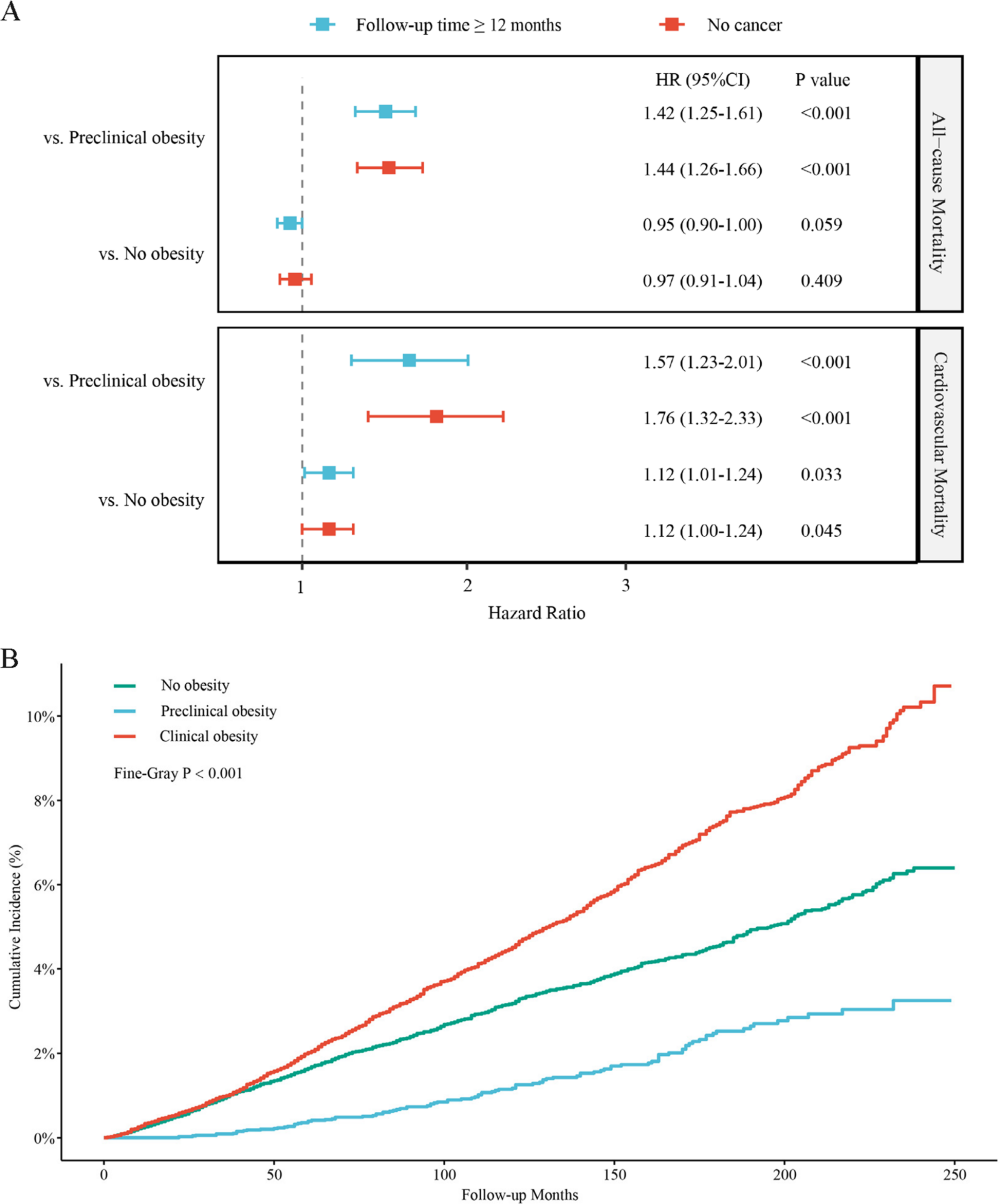
Sensitivity and competing risk analyses of obesity phenotypes and mortality. **A** Cox regression analyses from two sensitivity analyses of all-cause and cardiovascular mortality across obesity phenotypes: participants with follow-up time ≥ 12 months (*N =* 52,899; median follow-up 113 months) and participants with no cancer history (*N =* 48,565; median follow-up 115 months). **B** Fine-Gray competing risk model showing the cumulative incidence functions for cardiovascular mortality, accounting for non-cardiovascular death as a competing event, with a median follow-up time was 112 months. All models were adjusted for age, sex, race/ethnicity, PIR, education, smoking status, alcohol consumption, cancer history, and HEI-2015

The sensitivity analyses presented in Fig. 3A were conducted in two cohorts: participants with at least 12 months of follow-up and participants without cancer history. When clinical obesity was compared with preclinical obesity, both sensitivity analyses consistently demonstrated significantly elevated risks for all-cause mortality [(HR = 1.42, 95% CI: 1.25–1.61; *P <* 0.001) and (HR = 1.44, 95% CI: 1.26–1.66; *P <* 0.001)] and cardiovascular mortality [(HR = 1.57, 95% CI: 1.23–2.01; *P <* 0.001) and (HR = 1.76, 95% CI: 1.32–2.33; *P <* 0.001)]. Compared to no obesity, clinical obesity showed no significant difference for all-cause mortality in either sensitivity analysis [(HR = 0.95, 95% CI: 0.90–1.00; *P =* 0.059) and (HR = 0.97, 95% CI: 0.91–1.04; *P =* 0.409), respectively]. However, it was consistently associated with a higher risk of cardiovascular mortality [(HR = 1.12, 95% CI: 1.01–1.24; *P =* 0.033) and (HR = 1.12, 95% CI: 1.00–1.24; *P =* 0.045), respectively].

Furthermore, in the competing risk analysis for cardiovascular mortality (Fig. 3B), the CIFs, accounting for non-cardiovascular death as a competing event, differed significantly across obesity phenotypes (Fine-Gray test *P <* 0.001), with the clinical obesity group exhibiting the highest cumulative incidence.

### Comorbidity burden and mortality risk

**Fig. 4:**
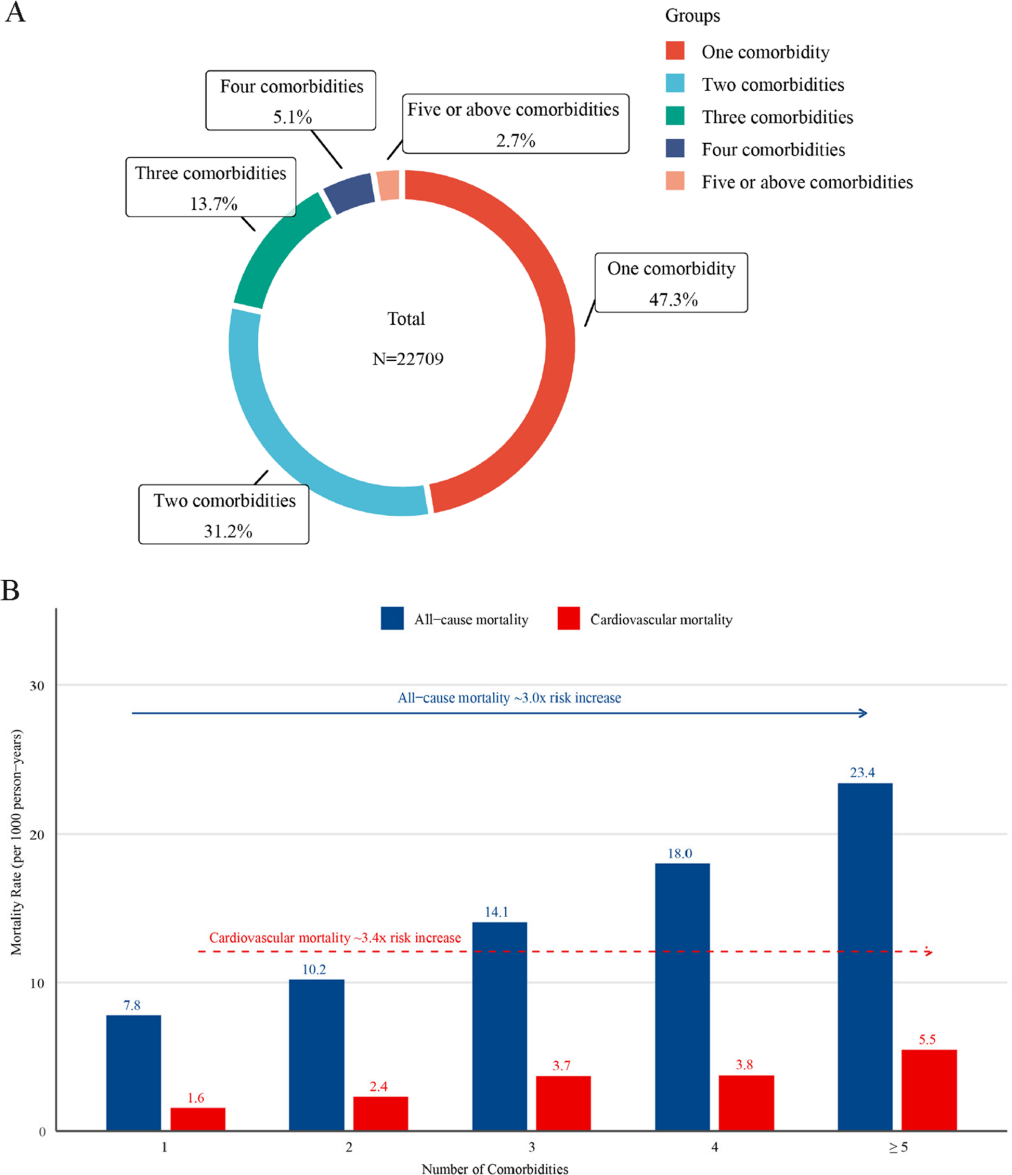
Comorbidity burden and dose–response relationship with mortality in clinical obesity. **A** Distribution of comorbidity burden. **B** Escalating mortality rates by multimorbidity count, with age- and sex-adjusted all-cause and cardiovascular mortality rates (per 1000 person-years) estimated using weighted Poisson regression

Within the clinical obesity group (*N =* 22,709), we further characterized the distribution of comorbidities and the impact of multimorbidity on survival outcomes. Overall, 47.3% of participants had only one comorbidity, whereas the remaining majority had two or more (Fig. 4A). As the number of comorbidities increased from one to more than five, the age- and sex-adjusted all-cause mortality rate rose from 7.8 to 25.4 deaths per 1000 person-years (approximately a 3.0-fold increase). Over the same range, the cardiovascular mortality rate increased from 1.6 to 5.1 deaths per 1000 person-years (approximately a 3.4-fold increase) (Fig. 4B).

### Phenotypic heterogeneity and prognostic divergence

**Fig. 5:**
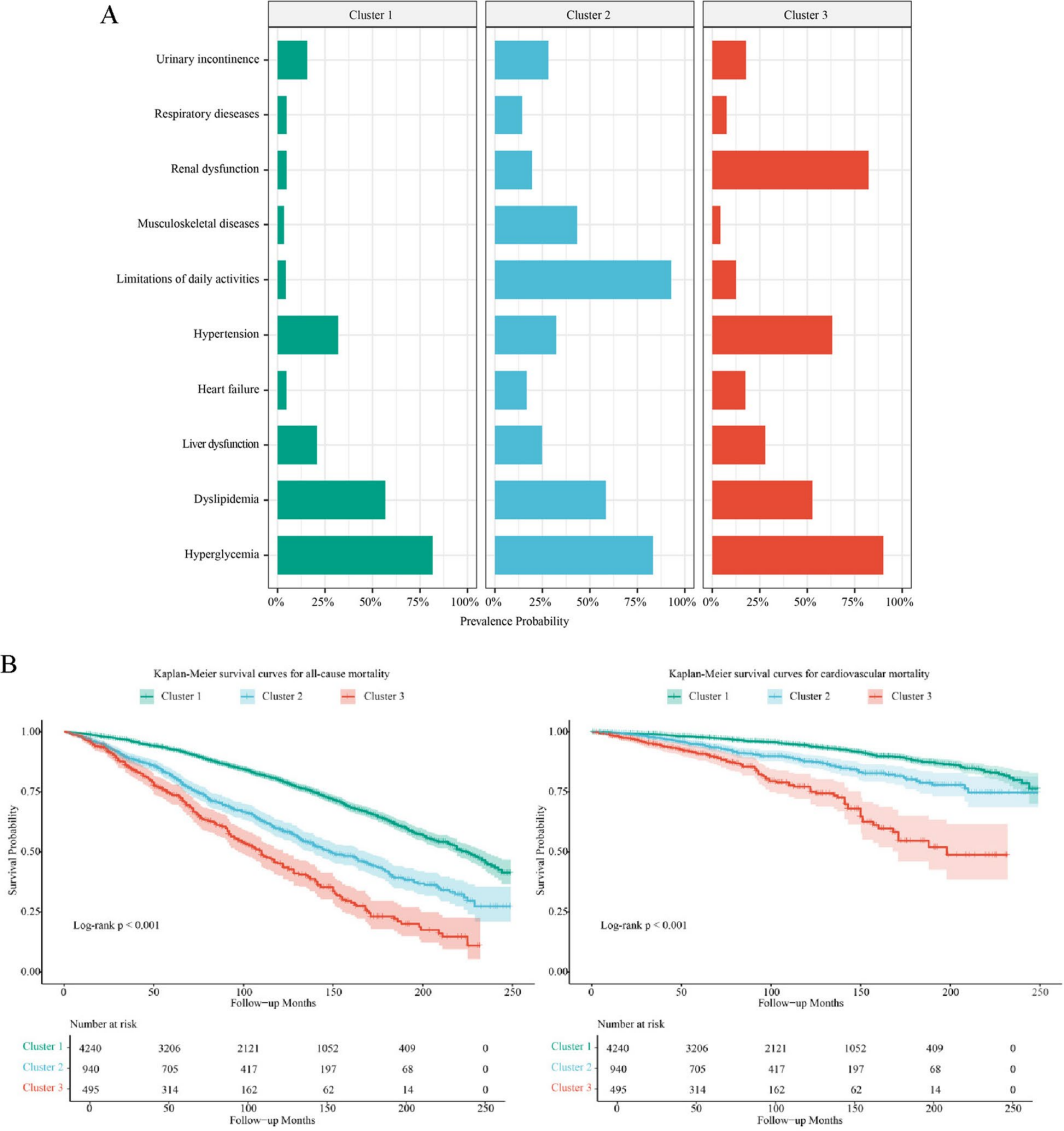
Identification and prognostic validation of comorbidity clusters within clinical obesity. **A** Latent class analysis-derived comorbidity clusters among participants with clinical obesity. **B** Kaplan–Meier survival curves for all-cause and cardiovascular mortality stratified by comorbidity clusters. Median follow-up time: 94 months

To further elucidate the risk of comorbidities, we employed LCA to identify three distinct phenotypic clusters within participants with clinical obesity (restricted to those without missing comorbidity data; Fig. 5A). Cluster 1 (*N =* 4240; 74.7%) mainly exhibited metabolic disorder characteristics, particularly a high probability of coexisting hyperglycemia, dyslipidemia, and hypertension. Cluster 2 (*N =* 940; 16.6%) demonstrated significant functional limitations beyond metabolic abnormalities, featuring daily activity limitations (> 90.0% prevalence) commonly accompanied by musculoskeletal diseases and urinary incontinence. Cluster 3 (*N =* 495; 8.7%) primarily featured high prevalence (> 80.0%) of renal dysfunction, together with hypertension, in addition to metabolic abnormalities.

Survival analyses showed that the three clusters followed markedly different survival trajectories (Fig. 5B). Compared with Cluster 1, Cluster 2 had a higher prevalence of multiple comorbidities—including respiratory disease, renal dysfunction, limitations in daily activities, musculoskeletal diseases, and urinary incontinence—and consequently a worse survival prognosis. Despite having a lower overall comorbidity burden, Cluster 3 experienced worse survival outcomes than Cluster 2, suggesting that renal dysfunction may exert a particularly adverse effect on prognosis. These findings underscore that mortality risk within the clinical obesity population is not uniform; rather, it is strongly stratified by specific comorbidity patterns, with the presence of renal dysfunction emerging as a critical determinant of adverse prognosis.

## Discussion

This study provides a nationally representative assessment of the prognostic significance of clinical obesity in US adults, based on the framework proposed by the Lancet Diabetes & Endocrinology Committee. Using NHANES data linked to mortality, we found that the clinical obesity phenotype was significantly associated with an increased risk of death, particularly cardiovascular mortality, and these associations were corroborated by sensitivity and competing risk analyses. Further analyses showed that the coexistence of renal dysfunction was associated with especially poor prognosis among individuals with clinical obesity. Taken together, these findings support the clinical utility of the newly proposed definition and underscore the importance of incorporating both comorbidity counts and specific comorbidity patterns into risk stratification for patients with clinical obesity.

Our data indicate a substantial burden of obesity among US adults, a finding that corroborates reports from the Global Burden of Disease Study [1]. With over 60.0% of the study population now classified as having obesity, our estimates align closely with recent findings from the All of Us research program, reinforcing the reliability of these results [9]. Consistent with emerging evidence [10, 11], clinical obesity constituted a major proportion of the total obesity burden, whereas preclinical obesity remained relatively stable. Stratified analyses revealed marked socioeconomic disparities in obesity phenotypes. Although the overall prevalence of obesity was broadly similar across socioeconomic groups, higher socioeconomic status (defined by education and income) was associated with a significantly lower likelihood of having clinical obesity compared with preclinical obesity [12, 13]. In contrast, the distribution of obesity phenotypes did not differ substantially across racial and ethnic groups, a finding that may be partly attributable to the aggregation of diverse minority populations in the analysis.

This phenotypic divergence suggests a structural shift within the population with obesity—likely potentially reflecting population aging and increasing disease severity—that collectively corresponds to a transition toward a more metabolically unhealthy state [14]. Consequently, the predominance of severe obesity phenotype disproportionately intensifies the strain on the healthcare system [15]. These findings underscore the need for targeted interventions aimed at mitigating the burden of clinical obesity, particularly in socioeconomically disadvantaged populations.

Our survival analysis unequivocally underscores the severe prognostic implications of this accumulating burden. Clinical obesity, compared to preclinical obesity, was associated with a 46.0% higher risk of all-cause mortality and a 65.0% higher risk of cardiovascular mortality. Notably, clinical obesity was also associated with a higher risk of cardiovascular mortality, even when compared to the no obesity group with comorbidities. Furthermore, interaction analyses showed that this excess mortality risk was concentrated in younger and middle-aged adults [16]. This marked divergence in survival trajectories corroborates findings from cohorts such as the UK Biobank [6], collectively highlighting the inadequacy of relying solely on BMI for comprehensive obesity evaluation [17]. This conceptual shift is further supported by the diagnostic framework from the European Association for the Study of Obesity, which likewise incorporates medical, functional, or psychological issues and complications within obesity diagnostic criteria [18]. However, while functional and organ dysfunction are central to these new obesity definitions, current frameworks often lack granular information regarding the severity of associated comorbidities [5, 18]. This gap underscores the critical necessity of characterizing the heterogeneity of obesity manifestations and staging the severity of its clinical presentation more comprehensively.

Our subsequent analysis within the clinical obesity population revealed significant heterogeneity in mortality risk. As the number of comorbidities increased from one to more than five, the risk of all-cause mortality escalated 3.0-fold, and cardiovascular mortality 3.4-fold [19, 20]. More critically, LCA showed that prognosis is determined not only by the number of comorbidities but also by the involvement of specific organ systems. Among patients with clinical obesity, those with renal dysfunction as the dominant feature had significantly lower survival rates compared to groups characterized primarily by metabolic abnormalities or functional impairment [21, 22]. The observed phenomenon strongly suggests that relying on a binary diagnosis of clinical obesity is insufficient for comprehensive risk assessment. Instead, clinical practice must actively prioritize screening for and intervening in patients who present with established dysfunction in critical organs such as the kidney and heart [23, 24]. These individuals often face a significantly bleaker prognosis compared to those with a high comorbidity burden but preserved organ function.

This study presents the first comprehensive evaluation of obesity phenotypes, defined by a novel diagnostic framework, and their prognostic significance within a nationally representative US cohort spanning two decades. Our findings advocate for a paradigm shift in obesity management, emphasizing that precise risk assessment necessitates integrating two critical dimensions: the cumulative burden of comorbidities and specific patterns of organ system involvement. This comprehensive stratification provides actionable prognostic insights essential for clinical decision-making, guiding optimized resource allocation and individualized interventions, especially for individuals with clinical obesity exhibiting established organ damage. However, the current framework remains subject to limitations, including ambiguous diagnostic criteria for hyperglycemia and dyslipidemia, and the exclusion of critical obesity-related factors such as atherosclerotic cardiovascular disease and mental health. Future iterations should integrate these elements to enhance diagnostic precision and clinical relevance.

Beyond the inherent limitations of the diagnostic framework itself, our study is subject to several methodological constraints. First, the cross-sectional design of the baseline assessment precludes the analysis of weight fluctuations or long-term adiposity trajectories. Second, the reliance on self-reported data for organ dysfunction and functional limitations introduces potential recall bias and lacks objective physician verification. Third, inconsistent data availability across NHANES cycles—particularly the absence of body fat quantification via dual-energy X-ray absorptiometry and the lack of granular comorbidity data (e.g., heart failure with preserved ejection fraction and sleep apnea)—likely resulted in a conservative estimation of obesity prevalence. Fourth, our analysis was constrained by the lack of adjustment for certain covariates, such as menopausal status (owing to inconsistent availability), as well as physical activity and cardiometabolic medication use (attributable to significant data missingness). Fifth, LCA was inherently limited by its cross-sectional nature, precluding the capture of longitudinal disease trajectories or assessment of cluster stability. Additionally, classification uncertainty was not propagated into subsequent survival models, rendering the identified clusters exploratory rather than definitive clinical phenotypes. Finally, while clinical guidelines often infer a causal relationship, the observational design of NHANES precludes definitive establishment of causality between obesity and comorbidities; moreover, potential bidirectional relationships and residual confounding by unmeasured. Consequently, classifying individuals solely based on the co-occurrence of these conditions as 'clinical obesity' may introduce a degree of misclassification bias.

Our study reveals that the expanding obesity epidemic in the US is predominantly characterized by a growing burden of high-risk clinical obesity, signifying a shift towards more severe disease phenotypes. Within this population, mortality risk escalates with the burden of comorbidities and varies significantly based on specific organ system involvement. Given the growing recognition of obesity as a chronic condition requiring sustained management, the quantification of comorbidity scores is imperative for refining risk stratification and guiding targeted therapeutic interventions.

## Supplementary Information

Supplementary Material 1.

Supplementary Material 2.

Supplementary Material 3.

## Data Availability

All the data are freely downloaded from the National Health and Nutritional Examination Survey (https://www.cdc.gov/nchs/nhanes).

## Abbreviations

BMI: Body mass index
NHANES: National Health and Nutrition Examination Survey
HR: Hazard ratio
LCA: Latent class analysis
PIR: Poverty-income ratio
SD: Standard deviation
HEI-2015: Healthy Eating Index-2015
