# Supplementary material for: Prevalence and comorbidity burden of clinical obesity in US adults
Source: BMC Public Health. 2026 Apr 29;26:1888. doi: 10.1186/s12889-026-27457-3 (PMC13274020; doi:10.1186/s12889-026-27457-3)
Supplement: Supplementary file 1 — Supplementary Material 1. [file 12889_2026_27457_MOESM1_ESM.docx]

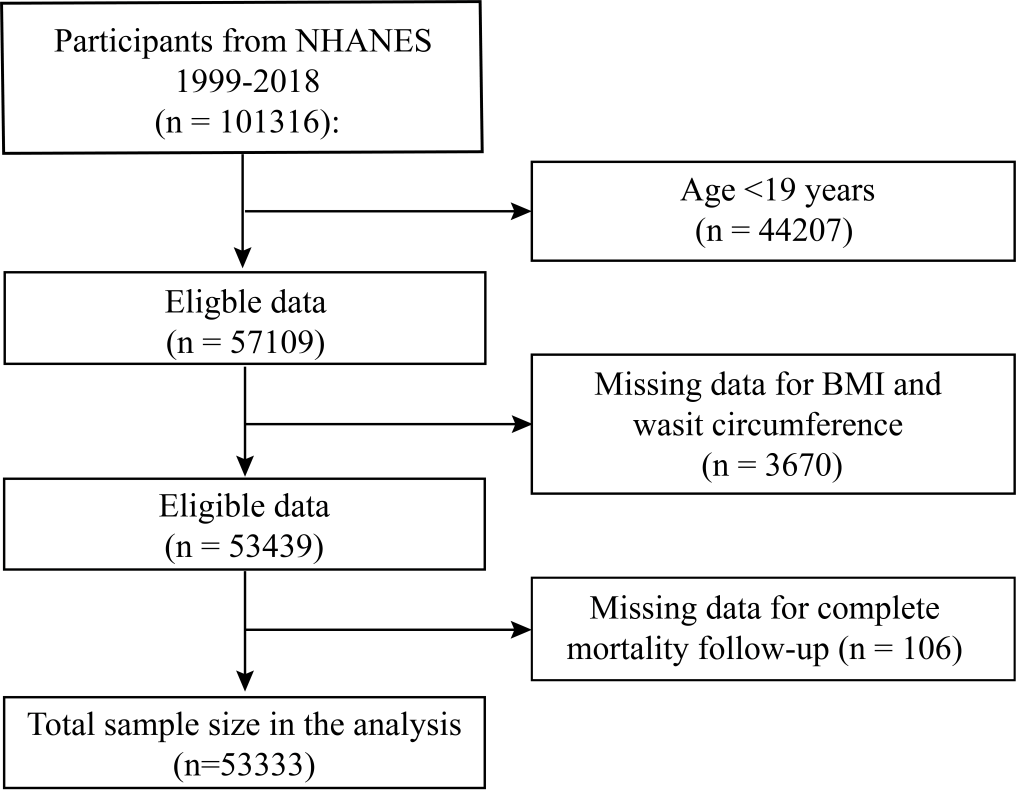


**Supplementary Figure 1** Flowchart of the study population

Supplementary **Table 1** Criteria for clinical obesity based on the Lancet Commission Definition

| **Obesity was defined as meeting any of the following three criteria** | | | | |
| --- | --- | --- | --- | --- |
| 1 | BMI ≥40 kg/m^2^ | | | |
| 2 | BMI plus waist circumference, or waist-to-height ratio | | | |
| 3 | Waist circumference and waist-to-height ratio | | | |
| Criteria by measurement | | Male | Female | |
| BMI | | ≥ 30 kg/m^2^ (Asian: ≥ 27.5 kg/m^2^ ) | ≥ 30 kg/m^2^ (Asian: ≥ 27.5 kg/m^2^ ) | |
| Waist circumference | | ≥ 102cm (Asian: ≥ 90 cm) | ≥ 88cm (Asian: ≥ 80 cm) | |
| Waist-to-height ratio | | > 0.5 | > 0.5 | |
| **Clinical obesity was defined as the presence of organ system dysfunction or impairments in mobility and activities of daily living** | | | | |
| Central nervous system | | Signs of raised intracranial pressure such as vision loss and/or recurrent headaches | | Not assessed |
| Upper airways | | Apnoeas/hypopnoeas during sleep due to increased upper airways resistance | | Not assessed |
| Respiratory | | Hypoventilation and/or breathlessness and/or wheezing due to reduced lung and/or diaphragmatic compliance | | Chronic obstructive pulmonary disease and emphysema (self-reported) |
| Cardiovascular (ventricular) | | Reduced Left Ventricular systolic function Heart Failure with Reduced Ejection Fraction - HFrEF | | Heart failure (self-reported) |
| Cardiovascular (atrial) | | Chronic/recurrent atrial fibrillation | | Not assessed |
| Cardiovascular (pulmonary) | | Pulmonary artery hypertension | | Not assessed |
| Cardiovascular | | Chronic fatigue, lower limb edema due to impaired diastolic dysfunction – Heart Failure with Preserved Ejection Fraction - HFpEF | | Not assessed |
| Cardiovascular (thrombosis) | | Recurrent DVT and/or pulmonary thromboembolic disease | | Not assessed |
| Cardiovascular (arterial) | | Raised arterial blood pressure | | Systolic blood pressure ≥ 140mmHg, or diastolic blood pressure ≥ 90mmHg. |
| Metabolism | | The cluster of hyperglycaemia, high triglyceride levels, and low HDL cholesterol levels | | Hyperglycaemia：   1. Glycated hemoglobin levels between 5.7% and < 6.5%; 2. Fasting plasma glucose levels between 101 mg/dL and 126 mg/dL; 3. A 2-hour OGTT blood glucose level ranging from 140 mg/dL to 200mg/dL.   Dyslipidemia:  1) HDL cholesterol <50/40 mg/dL in female/male.  2) Triglycerides ≥150 mg/dL. |
| Liver | | NAFLD with hepatic fibrosis | | Fatty Liver Index (FLI) ^#^≥ 60 with Fibrosis-4 Index(FIB-4)^#^ >1.3 (Age ≥ 65, FIB-4>2.0) |
| Renal | | Microalbuminuria with reduced eGFR | | Renal dysfunction: estimated glomerular filtration rate (eGFR)^*^ < 90 with ACR > 30mg/g |
| Urinary | | Recurrent/chronic urinary incontinence | | Difficulty controlling bladder or leak urine(self-reported) |
| Reproductive (female) | | Anovulation, oligo-menorrhea and PCOS | | Not assessed |
| Reproductive (male) | | Male hypogonadism | | Not assessed |
| Musculoskeletal | | Chronic, severe knee or hip pain associated with joint stiffness and reduced range of joint motion | | Difficulty with stooping, crouching, or kneeling (self-reported) |
| Lymphatic | | Lower limbs lymphedema causing chronic pain and/or reduced range of motion | | Not assessed |
| Limitations of day-to-day  activities | | Significant, age-adjusted limitations of mobility and/or other basic activities of daily living | | Difficulty with home-based activities, eating, walking, standing and sitting (self-reported) |
| * Estimated glomerular filtration rate (eGFR) was calculated using the following formula: eGFR = 142 × min(Scr/K, 1)^α^ × max(Scr/K, 1)^-1.200^ × 0.9938^Age^ × 1.012[if female]  Scr = standardized serum creatinine (mg/dL)  K = 0.7(females) or 0.9 (males)  α = -0.241 (females)or -0.302 (males)  min(Scr/K,1) is the minimum of Scr/K or 1  max(Scr/K,1) is the maximum of Scr/K or 1  Female if Creatinine ≤ 0.7mg/dL: eGFR = 142 x (Scr/0.7)-0.241 x 0.9938^Age^ x 1.012  Female if Creatinine > 0.7mg/dL: eGFR = 142 x (Scr/0.7)-1.200 x 0.9938^Age^ x 1.012  Male with Scr ≤ 0.9mg/dL: eGFR = 142 x (Scr/0.9)-0.302 x 0.9938^Age^  Male with Scr > 0.9mg/dL: eGFR = 142 x (Scr/0.9)-1.200 x 0.9938^Age^ | | | | |
| # Fatty Liver Index (FLI) was calculated using the following formula: FLI = (e^0.953∗ln(TG)+0.139∗BMI+0.718∗ln(GGT)+0.053∗WC−15.745^)/(1 + e^0.953∗ln (TG)+0.139∗BMI+0.718∗ln (GGT)+0.053∗WC−15.745^) ∗100.  Fibrosis-4 Index (FIB-4) was calculated using the following formula: FIB-4 = Age(years)×AST (U/L)/(PLT[10^9^/L]×√ALT [U/L]).  TG, Triglyceride; GGT, gamma-glutamyl transferase, AST, aspartate aminotransferase; ALT, alanine aminotransferase; PLT, platelet count. | | | | |

Supplementary Table 2 Estimation of age-specific hazard ratios (clinical obesity vs. no obesity)

| **Characteristics** | **Age (year)** | **HR (95% CI)** | **P value** | **P for Interaction** |
| --- | --- | --- | --- | --- |
| All-cause mortality | 30 | 1.37 (1.12-1.66) | 0.002 | <0.001 |
|  | 45 | 1.17 (1.03-1.33) | 0.018 |  |
|  | 60 | 1.00 (0.93-1.07) | 0.952 |  |
|  | 75 | 0.85 (0.81-0.90) | <0.001 |  |
| Cardiovascular mortality | 30 | 2.21 (1.36-3.61) | 0.001 | 0.003 |
|  | 45 | 1.70 (1.23-2.34) | 0.001 |  |
|  | 60 | 1.30 (1.10-1.53) | 0.002 |  |
|  | 75 | 1.00 (0.90-1.10) | 0.962 |  |
| Model: adjusted for sex, and race/ethnicity, PIR, education, smoking, alcohol consumption, cancer history, and HEI-2015. | | | | |

**Supplementary Table 3** Model characteristics and probabilities of latent class analysis

| **Clusters** | **Cluster size (N, %)** | **AvePP** | **Main high-probability comorbidities (P≈)** | |
| --- | --- | --- | --- | --- |
| Cluster 1 | 4240 (74.7%) | 0.949 | Hyperglycemia (0.82) | |
|  |  |  | Dyslipidemia (0.57) | |
|  |  |  | Hypertension (0.32) | |
| Cluster 2 | 940 (16.6%) | 0.756 | Limitations of daily activities (0.93) | |
|  |  |  | Hyperglycemia (0.83) | |
|  |  |  | Dyslipidemia (0.58) | |
| Cluster 3 | 495 (8.7%) | 0.736 | Hyperglycemia (0.90) | |
|  |  |  | Renal dysfunction (0.82) | |
|  |  |  | Hypertension (0.63) | |
| Abbreviations: AvePP, Average Posterior Probability; P≈, Item-response probability | | | |  |

**Supplementary Table 4** Hazard ratios for all-cause and cardiovascular mortality (preclinical obesity vs. no obesity)

| **Comparison** | **Outcome** | **Model** | **HR (95% CI)** | **P Value** |
| --- | --- | --- | --- | --- |
| Preclinical obesity vs. No obesity | All-cause mortality | Model 1 | 0.55 (0.48-0.62) | <0.001 |
|  |  | Model 2 | 0.61 (0.54-0.69) | <0.001 |
|  |  | Model 3 | 0.64 (0.56-0.72) | <0.001 |
|  | Cardiovascular mortality | Model 1 | 0.54 (0.42-0.70) | <0.001 |
|  |  | Model 2 | 0.64 (0.50-0.81) | <0.001 |
|  |  | Model 3 | 0.67 (0.53-0.87) | 0.002 |
| Model 1, unadjusted model; Model 2, adjusted for age, sex, and race/ethnicity; Model 3, adjusted for age, sex, and race/ethnicity, PIR, education, smoking, alcohol consumption, cancer history, and HEI-2015. | | | | |
